# Validity of Self‐Reported Data on Postpartum Haemorrhage

**DOI:** 10.1111/1471-0528.18359

**Published:** 2025-09-05

**Authors:** Annika W. Terpstra, Romy Bosvelt, Mallory D. Woiski, Marleen M. H. J. van Gelder

**Affiliations:** ^1^ IQ Health Science Department Radboud University Medical Center Nijmegen the Netherlands; ^2^ Department of Obstetrics and Gynecology Radboud University Medical Center Nijmegen the Netherlands

**Keywords:** obstetric haemorrhage, PRIDE Study, statistics: epidemiological surveys

Over the last decades, the incidence of postpartum haemorrhage (PPH) has increased in high‐income countries, which cannot be explained by known risk factors [1]. As PPH substantially contributes to maternal morbidity and mortality worldwide, identification of risk factors is warranted to halt this trend. Some registries and healthcare databases contain data on the amount of blood loss within 24 h postpartum that may be used for this purpose. In addition, PPH may be self‐reported in questionnaires in birth cohort studies. Two previous small‐scale studies, however, indicated that maternal recall of PPH is inaccurate [2, 3], but evidence on the validity of maternal self‐report on PPH using web‐based questionnaires is lacking. Therefore, we aimed to assess the validity of a self‐completed web‐based questionnaire on PPH within a birth cohort study.

This study used data collected in the PRIDE Study, an ongoing cohort study among pregnant women and their offspring in The Netherlands [4]. We included all PRIDE Study participants with an estimated date of delivery between January 2018 and December 2019 for whom data on PPH were available from both questionnaires and obstetric records (*N* = 799). PPH was assessed with a closed‐ended question requesting a single answer (blood loss after delivery: less than 500 mL, 500–1000 mL or more than 1000 mL) in the first postpartum questionnaire, which was administered two months after the estimated date of delivery. We calculated level of agreement, Cohen's kappa and measures of validity (sensitivity, specificity, positive predictive value [PPV], and negative predictive value [NPV]) for self‐reported PPH with cut‐off values of ≥ 500 mL and > 1000 mL with obstetric records as the reference standard for the total population, as well as stratified by level of education, parity and mode of delivery.

The results are presented in Table 1. Overall, values for agreement and validity measures were high (> 0.85) for both cut‐off values, with the exception of the PPV, which was 0.73 (95% confidence interval [CI] 0.67–0.78) for ≥ 500 mL and 0.71 (95% CI 0.60–0.81) for > 1000 mL. This was due to the relatively high number of false‐positive reports in relation to the number of true‐positive reports. For the PPH cut‐off value of ≥ 500 mL, women with a high level of education seemed to have higher agreement, sensitivity and PPV compared to women with a low/intermediate level of education (differences in validity measures > 0.05). We also observed differences in agreement, specificity and PPV by mode of delivery, with lower values for caesarean sections than for vaginal deliveries, as well as a higher sensitivity for primiparae. Cohen's kappa ranged between 0.48 (95% CI 0.30–0.67) for the PPH cut‐off value of ≥ 500 mL among women with a caesarean section to 0.83 (95% CI 0.76–0.91) for the PPH cut‐off value of ≥ 1000 mL among women with a high level of education.

**TABLE 1:** Reliability and validity measures for self‐report of postpartum haemorrhage through a web‐based questionnaire administered two months after the estimated date of delivery. Data from the PRIDE Study, 2018–2019.

PPH definition	No. of participants				Agreement (95% CI)	Kappa (95% CI)	Sensitivity (95% CI)	Specificity (95% CI)	PPV (95% CI)	NPV (95% CI)
	TP	FP	FN	TN						
≥ 500 mL	189	70	33	507	0.87 (0.85–0.89)	0.69 (0.64–0.75)	0.85 (0.80–0.90)	0.88 (0.85–0.90)	0.73 (0.67–0.78)	0.94 (0.92–0.96)
Level of education										
Low/intermediate	25	17	9	94	0.82 (0.75–0.88)	0.54 (0.38–0.69)	0.74 (0.57–0.86)	0.85 (0.77–0.91)	0.60 (0.44–0.74)	0.91 (0.85–0.96)
High	162	53	23	410	0.88 (0.86–0.91)	0.72 (0.67–0.78)	0.88 (0.82–0.92)	0.89 (0.85–0.91)	0.75 (0.69–0.81)	0.95 (0.92–0.97)
Parity										
Primiparous	126	44	14	250	0.87 (0.83–0.90)	0.71 (0.64–0.78)	0.90 (0.84–0.94)	0.85 (0.81–0.89)	0.74 (0.47–0.80)	0.95 (0.92–0.97)
Multiparous	63	26	19	257	0.88 (0.84–0.91)	0.66 (0.56–0.75)	0.77 (0.67–0.85)	0.91 (0.87–0.94)	0.71 (0.61–0.80)	0.93 (0.90–0.96)
Mode of delivery										
Vaginal	166	54	28	462	0.89 (0.86–0.91)	0.72 (0.66–0.78)	0.86 (0.80–0.90)	0.90 (0.87–0.92)	0.76 (0.70–0.81)	0.94 (0.92–0.96)
Caesarean section	21	16	5	44	0.76 (0.66–0.84)	0.48 (0.30–0.67)	0.81 (0.63–0.93)	0.73 (0.61–0.83)	0.57 (0.41–0.72)	0.90 (0.79–0.96)
> 1000 mL	51	21	0	727	0.97 (0.96–0.98)	0.82 (0.74–0.89)	1.00	0.97 (0.96–0.98)	0.71 (0.60–0.81)	1.00
Level of education										
Low/intermediate	4	3	0	138	0.98 (0.95–1.00)	0.72 (0.41–1.00)	1.00	0.98 (0.95–1.00)	0.57 (0.23–0.87)	1.00
High	47	17	0	584	0.97 (0.96–0.98)	0.83 (0.76–0.91)	1.00	0.97 (0.96–0.98)	0.73 (0.62–0.83)	1.00
Parity										
Primiparous	37	14	0	383	0.97 (0.95–0.98)	0.82 (0.73–0.91)	1.00	0.97 (0.94–0.98)	0.73 (0.59–0.84)	1.00
Multiparous	14	7	0	344	0.98 (0.96–0.99)	0.79 (0.64–0.94)	1.00	0.98 (0.96–0.99)	0.67 (0.45–0.84)	1.00
Mode of delivery										
Vaginal	45	17	0	648	0.98 (0.96–0.99)	0.83 (0.75–0.91)	1.00	0.97 (0.96–0.99)	0.73 (0.61–0.83)	1.00
Caesarean section	6	4	0	76	0.95 (0.90–0.99)	0.73 (0.47–0.98)	1.00	0.95 (0.89–0.98)	0.60 (0.30–0.85)	1.00

Abbreviations: CI, confidence interval; FN, false‐negative; FP, false‐positive; NPV, negative predictive value; PPH, postpartum haemorrhage; PPV, positive predictive value; TN, true‐negative; TP, true‐positive.

Our results showed that a web‐based questionnaire administered two months after the estimated date of delivery collects highly valid data on PPH, although overreporting is relatively frequent. These results are reassuring for studies on PPH using web‐based questionnaires as method of data collection. In addition, our estimates of sensitivity, specificity, PPV and NPV may be used as internal and external validation data to inform quantitative bias analyses to estimate uncertainty in effect estimates due to information bias in studies using self‐reported data on PPH as exposure, outcome or confounder [5]. It is important to acknowledge that the degree of overreporting differed by some maternal and pregnancy characteristics, providing the opportunity for bias of unpredictable size and direction when not taken into account in the analyses. Nevertheless, the validity of data on PPH collected with our web‐based questionnaire was substantially higher than previously reported for interviews, which administered subjective questions to assess PPH instead of attempting to objectively quantify the amount of blood loss [2, 3]. We conclude that datasets containing self‐reported data on PPH—if collected appropriately—may contribute to enhancing our understanding of risk factors for this common obstetric complication.

## Author Contributions

A.W.T. and M.M.H.J.G. conceived the study. A.W.T., R.B. and M.M.H.J.G. were responsible for data collection and analysis. All authors interpreted the results. A.W.T. and M.M.H.J.G. wrote the first draft of the manuscript, with revisions from R.B. and M.D.W. All authors approved the final manuscript.

## Ethics Statement

The PRIDE Study was approved by the MREC Oost‐Nederland (CMO 2009/305). All participants provided online informed consent.

## Conflicts of Interest

The authors declare no conflicts of interest.

## Data Availability

The data that support the findings of this study are available on request from the corresponding author. The data are not publicly available due to privacy or ethical restrictions.
